# Bumpy and Smoother Pathways of Puberty Hormone Change: A Novel Way to Define Gonadal Hormone Trajectories in Adolescents

**DOI:** 10.1210/jendso/bvz014

**Published:** 2019-11-29

**Authors:** Katharine S Steinbeck, Frances L Garden, Hoi Lun Cheng, Georgina M Luscombe, David J Handelsman

**Affiliations:** 1 The University of Sydney, Faculty of Medicine and Health, Sydney Medical School, Discipline of Child and Adolescent Health, Westmead, NSW, Australia; 2 The Children’s Hospital at Westmead, Academic Department of Adolescent Medicine, Westmead, NSW, Australia; 3 University of New South Wales, South Western Sydney Clinical School, Liverpool, NSW, Australia; 4 Ingham Institute for Applied Medical Research, Respiratory Medicine Research Stream, Liverpool, NSW, Australia; 5 The University of Sydney, Faculty of Medicine and Health, School of Rural Health, Orange, NSW, Australia; 6 The University of Sydney, ANZAC Research Institute, Concord, NSW, Australia

**Keywords:** puberty, adolescent, urine, testosterone, estradiol, longitudinal

## Abstract

**Context:**

The study of gonadal hormone effects on adolescent wellbeing has been limited by logistical challenges. Urine hormone profiling offers new opportunities to understand the health and behavioral implications of puberty hormones.

**Objective:**

To characterize pubertal change in urinary testosterone and estradiol among male and female adolescents, respectively.

**Design:**

Three-year prospective cohort study.

**Setting:**

Australian regional community.

**Participants:**

282 (163 male) normally developing adolescents aged 11.8 ± 1.0 years at baseline.

**Main outcome measure:**

Quarterly urine measurements of testosterone and estradiol (mass spectrometry); annual anthropometric assessment and Tanner stage (TS) self-report.

**Results:**

Two-class sigmoidal and quadratic growth mixture models (centered on age at TS3) were identified as best-fit for describing testosterone (male) and estradiol (female) change. Classes 1 (male: 63%; female: 82%) and 2 (male: 37%; female: 18%) were respectively named the “stable” and “unstable” trajectories, characterized by different standard deviation of quarterly hormone change and magnitude of hormone peaks and troughs (all *P* < 0.001). Compared with class 1 (stable), class 2 males were taller at baseline (154 vs 151 cm), reported earlier and faster TS progression (*P* < 0.01), and showed higher serum testosterone levels at baseline and 3 years (*P* ≤ 0.01). Class 2 females exhibited smaller height and weight gains over the 3 years and had higher baseline serum estradiol (249 vs 98 pmol/L; *P* = 0.002) than class 1.

**Conclusions:**

Adolescents showed 2 distinct urinary gonadal hormone trajectories, characterized by stability of change over time, which were not associated with consistent anthropometric differences. Results provide a methodology for studying gonadal hormone impacts on other aspects of biopsychosocial wellbeing. Identification of potential “at-risk” hormone groups would be important for planning supportive interventions.

## Introduction

Puberty, the transitional period between childhood and adulthood, is a remarkable physical event orchestrated by dramatic changes in gonadal and pituitary hormones. The 2 primary hormones of puberty, testosterone in males and estradiol in females, operate synergistically with growth hormone to produce an increase in body size and a transformation to the body to become physically and behaviorally capable of reproduction [1]. Other characteristic changes during this period include mood alterations, disengagement from education, and oppositional and other health risk behaviors, some of which are intermittent/transitory and some more permanent [1–3]. However, to date, the effect that gonadal hormones have on these other aspects of adolescent wellbeing and behavior remains unclear. Situations where gonadal hormones change rapidly, such as the menstrual cycle [4], pregnancy, and the postpartum period [5, 6], as well as in androgen abuse, are all linked to significant mood and behavior disturbances [7]. Thus, it is perhaps unsurprising that clinicians, parents, and the community commonly attribute adolescent mood and behavior change to puberty hormones, along with other influencing factors, such as neurocognitive development [8] and relationships with peers and family [9].

Understanding the true impact of gonadal hormones on adolescent health and wellbeing is important for 3 reasons. First, there is a large body of evidence demonstrating that off-timed pubertal onset (earlier or later) predicts long-term health including depression and metabolic disease risk [10, 11], indicating a potential hormonal mechanism. Tempo or the speed of change of puberty hormones might also be associated with mood or behavior alterations [12], although this research area is in its infancy and existing studies generally define tempo using proxies rather than direct gonadal hormone measurement [13, 14]. Second, the widespread distribution of gonadal hormone receptors, including in the brain [15, 16], makes the possibility of impacts outside the reproductive system very likely. Third, erroneous attribution of puberty hormone effects may draw attention away from other modifiable risk factors and result in missed opportunities for effective therapy/intervention. For example, the speculation that testosterone heightens antisocial or aggressive behaviors in adolescence is not supported by currently available data [17]. Such behaviors may instead be related to lack of learned impulse control or parental modeling, factors that are directly modifiable.

Studies seeking to examine the health and behavioral implications of puberty hormone change should directly measure relevant hormones, although such studies are challenging. Circulating testosterone rises in males from childhood levels by 20- to 30-fold over 2 to 5 years, while serum estradiol in females increase by 4- to 9-fold [18, 19]. Marked inter-individual variation in these hormones are observed across the physical stages of maturation [20–22]. These observations indicate that a high frequency of biological sampling is required to profile *individual* patterns/trajectories of testosterone and estradiol change, which is constrained by participant acceptability and resource limitations. Common practice has been to use infrequent blood sampling or proxies such as Tanner stage (TS), somatic growth parameters such as peak height velocity (PHV) and pubertal milestones, such as menarche and voice-break [10, 23-25]. On these measures, it is well recognized that significant inter-individual variation exists in timing and duration of puberty [26, 27]. More recently, the measurement of gonadal hormones in urine has been explored [28, 29]. Such research indicates advantages of urinalysis as a less invasive alternative to blood sampling and as a more detailed and temporally-integrated description of individual hormone trajectory than annual/biannual measurement of blood hormones [28].

The aim of this study was to examine pubertal change in urinary testosterone and estradiol in a sample of healthy adolescent males and females, respectively, followed over 3 years. The hypothesis was that individuals will show varying gonadal hormone trajectories, in particular, differences in the rate at which these hormones change. A secondary aim was to present novel insights and knowledge on longitudinal urinary gonadal hormone change in both sexes, given that we were unable to identify a previous study with such intensive and lengthy biological sampling.

## 1. Materials and Methods

### A. Study Setting and Participants

The protocol and baseline characteristics of the Adolescent Rural Cohort study of Hormones, Health, Environments, Education and Relationships (ARCHER) are published [30, 31]. In brief, the ARCHER study was designed to investigate the impact of puberty hormones on adolescent health, wellbeing, and behavior. Participants were recruited through schools and community groups from 2 regional cities and their vicinities in the state of New South Wales, Australia [32]. A total of 400 participants, allowing for 30% attrition, was needed for the study to be adequately powered. This was achieved with enrollment of 342 adolescents and an 82% retention rate at the end of 3 years. Given the intensity of the study protocol, 3 years was the maximum duration considered possible, based on feedback from initial feasibility studies and prior research experience in adolescents [33, 34]. A 3-year study duration would allow adolescents to reach at least mid-puberty (commonly described by TS3), which is when a noticeable upswing in gonadal hormone levels is evident [19, 35, 36], and when typical adolescent mood and behaviors become more prevalent [1, 30, 37]. Inclusion criteria were school grades fifth to seventh, capturing mostly ages 10 to 12 years at enrollment, and English proficiency for the completion of questionnaires on personal, family, and environmental information. These data, together with measured anthropometry and self-reported Tanner stage were collected annually as described elsewhere [30, 38]. Adolescents were asked to rate their pubertal status against standardized line drawings of genital and breast development for males and females, respectively (with no descriptive text) [26, 27]. Females were asked every quarter if/when they had reached menarche.

### B. Ethics

The ARCHER study was approved by the Human Research Ethics Committee of The University of Sydney (HREC 2010/13094 and 2015/199). Written informed consent was obtained from a parent/guardian prior to study commencement, together with adolescent assent.

### C. Specimen Collection and Biochemical Analyses

Fasting, first-morning urine samples (cycle days 7-10 in postmenarcheal females) were collected every 3 months over 3 years for the measurement of testosterone and estradiol. Fasting, morning serum samples were collected annually and contemporaneously with the corresponding urine sample over the 3 years, with the same menstrual cycle considerations. All biological samples were stored at −80 °C (−112 °F) across multiple aliquots. Stability of samples under such storage conditions has been demonstrated previously [39, 40]. Liquid chromatography tandem mass spectrometry was used to measure urine and serum testosterone and estradiol concentration. The methodology was modified from a protocol for analyzing serum [41] and adapted for urine specimens after enzymatic deconjugation with full assay performance characteristics published [28]. Urine specific gravity (SG) was estimated via reagent strip (ChoiceLine 10, Roche Diagnostics), with dipstick color changes compared visually against a SG color chart. All urine hormone concentrations were adjusted to a standard SG of 1.020 using the equation: hormone concentration_sample_ × (1.020−1) ÷ (SG_sample_−1) [29, 42]. Hydration status was not found to significantly influence hormone results in this study [29]. Urine testosterone (*r* = 0.77; *P* < 0.001) and estradiol (*r* = 0.72; *P* < 0.001) were correlated with but higher than serum levels [28]. As such, both urine hormones are expressed in nmol/L from here on, whereas serum hormone levels are presented in SI units. Conversion from SI to conventional units: testosterone nmol/L ÷ 3.467 = ng/mL; estradiol pmol/L ÷ 3.671 = pg/mL.

### D. Statistical Analysis

Growth mixture models (GMM) were employed to identify varying trajectories of urinary hormone change in each sex. Briefly, GMM is a data-driven analytic approach that has the capacity to handle complex and variable change (in both positive and negative directions) in longitudinal data, which enabled us to quantifiably describe urinary hormone patterns over time in individuals [43]. GMMs allow identification of (latent) classes that are defined by their patterns of change, with no predetermination of class number, type, or pattern. The advantage of GMMs over conventional analyses of change using random effects models is that GMMs do not rely on an assumption that all participants of the cohort come from the same underlying distribution. Hence, GMMs are more flexible and robust than conventional approaches to analyzing heterogeneous longitudinal data such as the urine hormones in this study. Specifically, GMMs test the hypothesis that there are subgroups of individuals who share a common hormone trajectory, but the hormone trajectories differ between groups. The general pattern of hormone change identified, by preliminary plots, was an increase over time to a level higher than baseline in both sexes. However, sex-specific plots of individual trajectories did not show a clear polynomial shape/trend over time. Thus, within the GMM framework various polynomial, semiparametric, and nonparametric shapes were tested. To determine the optimal number of hormone classes, a series of sex-specific GMMs with 1 to 3 classes were constructed for each functional form. The following criteria were used to determine the optimal number of classes: (i) convergence; (ii) Bayesian Information Criterion (BIC); (iii) adjusted BIC (aBIC); (iv) Akaike’s Information Criterion (AIC); (v) entropy; (vi) class size; and (vii) interpretability [44]. Each individual within the cohort is allocated to the class for which they had the highest probability of membership. Our input variables for GMM were individual urine testosterone or estradiol concentrations (for males and females, respectively) and centered age (treated as the time exposure variable). Age alone is not a consistent marker of puberty. Hence, the chronological age corresponding to each urine sample was centered on the participant’s age at TS3, so as to standardize individual hormone trajectories to a common level of physical maturity. Age at TS3 was selected as the centering value common to both sexes, representing both the midpoint of puberty and the stage identified in previous studies as marking the acceleration of gonadal hormone increase [18, 45].

For completeness, age at menarche in females and age at PHV in both sexes were tested as alternate centering values. These alternate centering strategies had limitations, namely the study was powered on the assumption that the majority of participants would reach at least Tanner stage 3, and height was assessed annually with PHV assigned to the midpoint age between 2 annual height measures. Given the 3-year duration of the study, PHV was missed (male: n = 21; female: n = 52) or not yet reached (male: n = 35; female: n = 7) in some participants. Similarly, not all girls had reached menarche. Full details of our analytic strategy, including decisions for participant inclusion and model selection, can be found in the online supplementary material [46]. Mplus version 7.3 was used for growth mixture modeling [47].

Independent sample *t*-tests were used to assess differences in age, anthropometry, timing and tempo of TS progression, male serum testosterone (log transformed) and female serum estradiol (log transformed) between included and excluded participants and between classes obtained from GMM. The same tests were applied to compare urine hormone trajectory characteristics between GMM classes. Timing and tempo of TS progression were derived mathematically for each individual using published methods involving linear and logistic mixed modeling of annual self-reported TS [48, 49]. Results are provided as mean ± standard deviation (SD) or median (interquartile range), with statistical significance set at *P* < 0.05. SAS Version 9.4 (SAS Institute, Cary, NC) was used for this set of analyses.

## 2. Results

**Figure 1.**
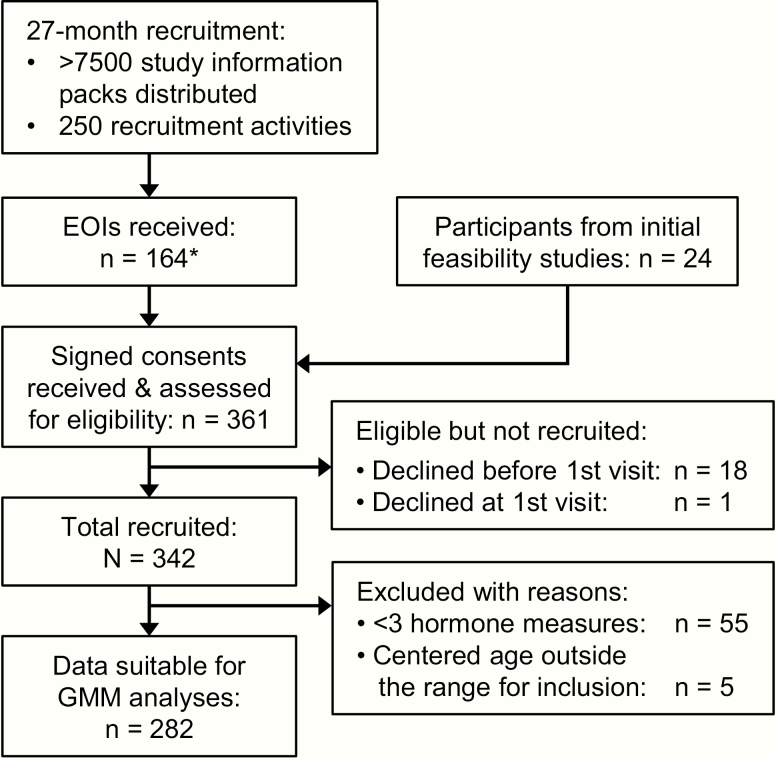
Flow chart outlining the ARCHER study recruitment process and exclusion of participants from growth mixture modeling. *Submission of an EOI was not essential for recruitment into the study. (EOIs, expressions of interest)

Of the total 342 participants recruited to the ARCHER study, 282 (163 males, 119 females) were included in the GMM analyses (Fig. 1). These participants provided an average of 9.7 urine hormone measures (out of a maximum 13) over the 3 years. Baseline age was 11.9 ± 1.0 years for males and 11.7 ± 0.9 years for females. Comparison of participants included and excluded from GMM analyses showed no age, anthropometric, baseline serum estradiol, or TS differences among females (Table S1) [46]. Excluded males were identified as the later developers of the cohort, exhibiting lower TS (baseline: 2.1 vs 2.7; 3 years: 2.3 vs 4.5; both *P* < 0.01) and serum testosterone levels (baseline: 0.04 vs 1.1 nmol/L; 3 years: 1.7 vs 10.3 nmol/L; both *P* < 0.01) (Table S1) [46]. Height change over the study duration was also smaller among excluded males (14.1 vs 19.6 cm; *P* = 0.018). A PHV year was evident in 110 males and 58 females.

**Figure 2.**
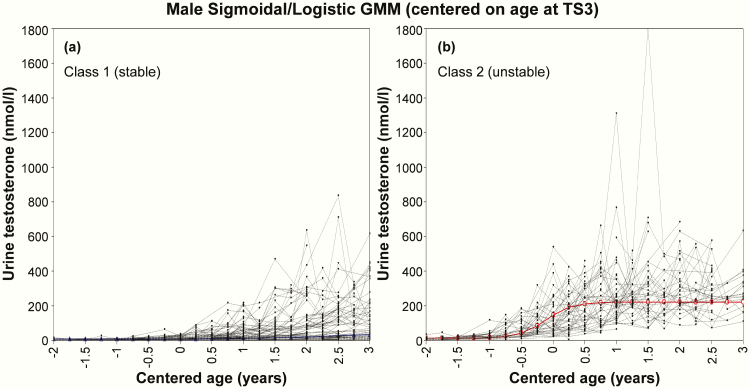
Final 2-class sigmoidal/logistic model, centered on age at Tanner Stage 3, which show a **(a)** stable and **(b)** unstable pattern of urinary testosterone change in males. Colored lines denote the average trajectory among participants in that class.

**Figure 3.**
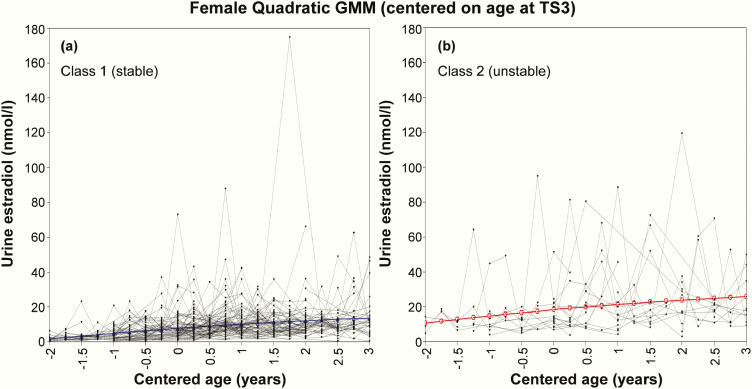
Final 2-class quadratic model, centered on age at Tanner Stage 3, which show a **(a)** stable and **(b)** unstable pattern of urinary estradiol change in females. Colored lines denote the average trajectory among participants in that class.

Comparison of model-fit statistics between various GMMs showed that, regardless of centering value, urinary testosterone and estradiol change in males and females were best described by sigmoidal/logistic and quadratic 2-class models, respectively (Table 1). Among the 2 classes identified by the TS3-centered GMMs, class 1 contained 63% (n = 103) of males and 82% (n = 98) of females, and was arbitrarily named the “stable class” for a more consistent and steady rise in urine hormone over 3 years. Class 2, containing 37% (n = 60) of males and 18% (n = 21) of females, was named the “unstable class” due to a pattern of urinary hormone rise characterized by greater fluctuation and higher overall levels across the study duration. These patterns were apparent upon observing the male (Fig. 2) and female (Fig. 3) GMM plots and were subsequently quantified through calculation of hormone trajectory characteristics (Table 2). Centering on the late pubertal event of menarche resulted in 77% (82/106) of females being allocated to class 1 (Table S2; Fig. S1) [46]. Alternatively, centering on age at PHV yielded a class 1 size of 94% (103/110) among males and 86% (50/58) among females (Table S3; Fig. S2) [46]. A complete set of model-fit statistics (Tables S4-S8), including considerations for 3-class models and plots for several alternative curve shapes (Figs S3-S4), are available elsewhere [46].

**Table 1.** Comparison of Model-Fit Statistics for Various 2-Class Growth Mixture Models Used to Describe Urine Testosterone and Estradiol Change in Males and Females, Respectively

Model Description	Parameters	AIC	BIC	aBIC	Entropy^a^	Class 1 n (%)	Class 2 n (%)
**Urine testosterone, centered on age at TS3**							
Sigmoidal/logistic (2-class; best fit)	36	15777	15888	15774	0.760	103 (63.2)	60 (36.8)
Gompertz (2 class)	36	15840	15951	15837	0.773	113 (69.3)	50 (30.7)
Cubic (2-class)	30	16242	16335	16240	0.927	109 (66.9)	54 (33.1)
Quadratic (2-class)	28	16329	16415	16327	0.931	101 (61.9)	62 (38.0)
**Urine testosterone, centered on age at PHV**							
Sigmoidal/logistic (2-class; best fit)	36	11759	11856	11742	0.970	103 (93.6)	7 (6.3)
Gompertz (2 class)	36	11764	11862	11748	0.914	105 (95.5)	5 (4.5)
Cubic (2-class)	30	12346	12427	12332	0.990	103 (93.6)	7 (6.3)
Quadratic (2-class)	28	12402	12478	12390	0.986	103 (93.6)	7 (6.3)
**Urine estradiol, centered on age at TS3**							
Quadratic (2-class; best fit)	28	8031	8109	8020	0.851	98 (82.4)	21 (17.6)
Cubic (2-class)	30	8032	8115	8020	0.862	99 (83.2)	20 (16.8)
Linear (2-class)	26	8029	8102	8020	0.845	100 (84.0)	19 (16.0)
**Urine estradiol, centered on age at menarche**							
Quadratic (2-class; best fit)	32	7250	7335	7234	0.798	82 (77.4)	24 (22.6)
Cubic (2-class)	34	7251	7341	7234	0.802	81 (76.4)	25 (23.6)
Linear (2-class)	30	7278	7358	7263	0.801	86 (81.1)	20 (18.9)
**Urine estradiol, centered on age at PHV**							
Quadratic (2-class; best fit)	24	3958	4007	3932	0.954	50 (86.2)	8 (13.8)
Cubic (2-class)	26	3944	3997	3915	0.954	50 (86.2)	8 (13.8)
Linear (2-class)	22	3967	4013	3944	0.936	50 (86.2)	8 (13.8)

Abbreviations: AIC, Akaike’s Information Criterion; aBIC, sample size adjusted Bayesian Information Criterion; BIC, Bayesian Information Criterion; PHV, peak height velocity; TS, Tanner stage.

^a^Entropy quantifies the uncertainty with which participants were categorized into latent classes, where 0 = random and 1 = perfect fit.

**Table 2.** Comparison of Hormone Trajectory Characteristics Between The 2 Urine Hormone Classes Identified by the TS3-Centered Growth Mixture Models

	Male Urine Testosterone Classes (centered on age at TS3)			Female Urine Estradiol Classes (centered on age at TS3)		
Hormone Trajectory Characteristics	Class 1 (stable)	Class 2 (unstable)	*P* value	Class 1 (stable)	Class 2 (unstable)	*P* value
N	103	60		98	21	
Study participation duration, y	2.9 (0.4)	2.9 (0.4)	0.839	2.8 (0.5)	2.6 (0.7)	0.297
Number of hormone measures	9.9 (2.1)	9.5 (2.1)	0.191	9.9 (2.8)	8.0 (2.7)	**0.010**
Absolute hormone concentration, nmol/L						
*At baseline*	17.0 (32.2)	77.7 (111.6)	**<0.001**	4.4 (4.8)	11.7 (7.3)	**0.001**
*At 3 years*	134.2 (141.8)	330.1 (200.4)	**<0.001**	12.5 (11.0)	21.7 (12.1)	**0.003**
*Median over 3 years*	52.4 (64.5)	167.5 (109.6)	**<0.001**	7.0 (3.7)	15.7 (5.1)	**<0.001**
*Lowest during study*	13.9 (26.3)	51.7(55.1)	**<0.001**	2.6 (2.6)	6.6 (3.3)	**<0.001**
*Highest during study*	163.3 (176.1)	451.4 (289.8)	**<0.001**	24.6 (21.3)	60.9 (24.2)	**<0.001**
Hormone change (any direction), nmol/L						
*Change from baseline to 3 years*	116.8 (135.6)	252.4 (233.0)	**<0.001**	7.7 (11.0)	9.5 (12.5)	0.546
*Change per year*	42.3 (48.5)	88.4 (80.1)	**<0.001**	2.9 (4.4)	4.8 (6.6)	0.241
*Mean quarterly change*	13.9 (16.3)	32.6 (38.1)	**<0.001**	1.1 (1.5)	1.8 (2.2)	0.154
*Median quarterly change*	9.0 (15.3)	25.3 (55.1)	**<0.001**	0.4 (1.8)	0.7 (4.0)	0.744
*SD of quarterly change*	44.0 (61.7)	151.5 (126.2)	**<0.001**	9.5 (11.0)	29.7 (15.8)	**<0.001**
Max. and min. quarterly hormone change, nmol/L						
*Max. quarterly change (any direction)*	93.6 (115.8)	300.2 (223.6)	**<0.001**	18.7 (20.9)	50.3 (24.2)	**<0.001**
*Min. quarterly change (any direction)*	3.5 (6.9)	12.5 (23.6)	**0.005**	0.7 (1.1)	2.6 (2.9)	**0.014**
*Max. quarterly peak*	90.5 (109.9)	285.0 (211.8)	**<0.001**	17.6 (20.9)	46.6 (25.7)	**<0.001**
*Min. quarterly peak*	4.9 (8.7)	24.6 (46.1)	**0.002**	1.5 (2.2)	8.8 (16.9)	0.058
*Max. quarterly trough*	−51.7 (96.7)	−211.1 (232.3)	**<0.001**	−13.6 (19.8)	−44.1 (22.4)	**<0.001**
*Min. quarterly trough*	−12.1 (26.0)	−33.3 (50.3)	**0.004**	−2.2 (2.9)	−7.3 (12.8)	0.086

Data are presented as mean (SD). All *P* values below 0.05 are shown in bold text. Abbreviations: Max, maximum; Min, minimum; TS, Tanner stage.

Compared with class 1 (stable), adolescents allocated to class 2 (unstable) in the TS3-centered GMMs showed significantly greater SD of quarterly testosterone/estradiol change (male: 151.5 vs 44.0; female: 21.3 vs 7.0 nmol/L; both *P* < 0.001) (Table 2). Class 2 adolescents also exhibited significantly larger maximum quarterly hormone peaks (male: 285.0 vs 90.5; female: 32.3 vs 13.2 nmol/L; both *P* < 0.001) and troughs (male: −211.1 vs −51.7; female: −26.8 vs −9.9 nmol/L; both *P* < 0.001) over the study duration. Median hormone level across 3 years was 2- to 3-fold higher in class 2 compared with class 1 adolescents (male: 167.5 vs 52.4; female: 13.2 vs 6.6 nmol/L; both *P* < 0.001). Absolute change in urine hormone level from baseline to 3 years was significantly greater among class 2 males (252.4 vs 116.8 nmol/L; *P* < 0.001) but not females (Table 2). Similar hormone trajectory differences were evident between the 2 menarche-centered GMM classes (Table S2) but not the PHV-centered classes (Table S3) [46]. Notably, the menarche-centered plots showed some premenarcheal spikes in urinary estradiol concentration that were similar in magnitude to those observed postmenarche (Fig. S1). This observation was supported statistically by the absence of a significant difference in premenarcheal (11.7 ± 14.7 nmol/L) versus postmenarchal (15.1 ± 15.8 nmol/L) maximum quarterly estradiol peaks (*P* = 0.35), irrespective of class.

A comparison of physical growth and maturation characteristics between the TS3-centered hormone trajectory classes is presented in Table 3. Class 2 (unstable) males showed significantly taller baseline height (*P* = 0.048) and earlier age at maximal height change (*P* = 0.005). Serum levels of testosterone among class 2 males were also significantly higher at baseline (*P* < 0.001) and at 3 years (*P* = 0.010). Class 2 males reported a significantly lower TS at baseline (2.5 vs 2.9; *P* = 0.033), despite TS3 centering, but not at 3 years (*P* = 0.079). This difference translated into significantly later timing and faster tempo of puberty (estimated from linear and logistic modeling of TS progression) among class 2 males (all *P* < 0.01).

**Table 3.** Descriptive Characteristics of Participants in the 2 Urine Hormone Classes Identified By the TS3-Centered Growth Mixture Models

	Male Urine Testosterone Classes (centered on age at TS3)					Female Urine Estradiol Classes (centered on age at TS3)				
	Class 1 (stable)		Class 2 (unstable)			Class 1 (stable)		Class 2 (unstable)		
Characteristic	n	Mean (SD)	n	Mean (SD)	*P* value	n	Mean (SD)	n	Mean (SD)	*P* value
Age, y										
*At baseline*	103	11.8 (1.0)	60	12.0 (1.0)	0.125	98	11.6 (0.9)	21	11.8 (1.1)	0.351
*At 3 years*^a^	97	14.7 (1.0)	56	15.0 (1.0)	0.095	90	14.7 (0.9)	19	14.9 (1.1)	0.276
Serum hormone concentration^b,c^										
*At baseline*	93	0.6 (0.1, 4.9)	51	3.4 (0.8, 13.0)	**<0.001**	89	97.6 (55.1, 175.5)	19	248.6 (139.1, 287.8)	**0.002**
*At 3 years*	75	9.8 (6.0, 12.6)	42	11.2 (9.3, 13.3)	**0.010**	54	114.2 (77.1, 161.5)	13	186.9 (82.2, 230.2)	0.102
Age at menarche, y	-	-	-	-	-	86	12.9 (1.5)	18	12.5 (1.1)	0.334
Tanner stage										
*At baseline*	103	2.9 (1.0)	59	2.5 (1.2)	**0.033**	96	2.3 (1.0)	21	2.7 (1.1)	0.194
*At 3 years*	96	4.4 (0.9)	56	4.7 (0.6)	0.079	90	4.2 (0.8)	19	4.2 (0.8)	0.786
*Maximum TS*	103	4.5 (0.7)	60	4.7 (0.6)	**0.047**	98	4.3 (0.8)	21	4.2 (0.7)	0.925
*Linear timing (age at TS3)*^d^	103	11.9 (1.1)	60	12.7 (1.0)	**<0.001**	98	12.3 (1.1)	21	12.4 (1.1)	0.802
*Linear tempo (average ∆TS/y)*^d^	103	0.5 (0.3)	60	0.7 (0.4)	**0.004**	98	0.7 (0.4)	21	0.5 (0.4)	0.133
*Logistic timing (age at TS3)*^e^	103	12.3 (0.8)	60	12.6 (0.7)	**0.004**	98	12.6 (0.8)	21	12.5 (0.9)	0.646
*Logistic tempo (slope at TS3; TS/y)*^e^	103	1.0 (0.4)	60	1.2 (0.4)	**0.002**	98	0.9 (0.2)	21	0.8 (0.2)	0.188
Height, cm										
*At baseline*	103	151.1 (10.2)	60	154.4 (10.0)	**0.048**	98	150.1 (8.9)	21	153.7 (8.0)	0.093
*At 3 years*	97	170.2 (9.6)	55	173.7 (6.8)	0.992	89	163.9 (6.6)	17	163.6 (4.8)	0.856
*∆ over 3 years*	97	19.4 (5.0)	55	20.0 (5.5)	0.494	89	14.0 (5.5)	17	10.3 (4.5)	**0.015**
*Maximum ∆ height, cm/y*	103	8.8 (1.9)	60	9.2 (2.2)	0.176	98	6.7 (2.6)	21	5.7 (2.5)	0.114
*Age at maximum ∆ height, y*	66	13.7 (1.0)	44	13.1 (0.9)	**0.005**	49	12.2 (0.8)	6	11.5 (0.6)	**0.048**
Weight (kg)										
*At baseline*	103	44.0 (12.7)	60	46.4 (11.3)	0.243	98	44.8 (12.1)	21	48.5 (12.4)	0.218
*At 3 years*	97	62.0 (16.6)	56	66.3 (13.8)	0.107	89	60.3 (13.1)	17	58.5 (11.4)	0.578
*∆ over 3 years*	97	18.3 (6.3)	56	20.0 (7.2)	0.134	89	16.1 (7.1)	17	11.2 (5.9)	**0.012**

*P* values below 0.05 are shown in bold text. Abbreviations: ∆, change; SD, standard deviation; TS, Tanner stage.

^a^Reduced sample size for measures at 3 years are due to study attrition and adolescent refusal to provide a blood sample.

^b^Data presented as median (interquartile range).

^c^Serum testosterone in males expressed in nmol/L; serum estradiol in females expressed in pmol/L.

^d^Timing and tempo derived from linear models of Tanner stage change (Beltz *et al*. Dev Psychol 2014;50:2715–26).

^e^Timing and tempo derived from logistic models of Tanner stage change (Marceau *et al.* Dev Psychol 2011;47:1389–409).

Based on the TS3-centered GMM (Table 3), females in class 2 (unstable) exhibited significantly smaller height (10.3 vs 14.0 cm; *P* = 0.015) and weight (11.2 vs 16.1 kg; *P* = 0.012) gain over 3 years. However, absolute measures of height and weight were similar to class 1 at baseline and 3 years. Class 2 females had higher serum estradiol levels at baseline (248.6.0 vs 97.6 pmol/L; *P* = 0.002), with no differences at 3 years (*P* = 0.102). Age at menarche, together with the timing and tempo of TS progression were similar between the TS3-centered classes. Comparison of participants allocated to the 2 menarche- and PHV-centered classes showed no physical maturation or serum estradiol differences (Tables S9-S10) [46].

## 3. Discussion

This study used more frequent biological sampling than many other longitudinal studies of puberty which address gonadal hormone change. A research protocol of such intensity was necessary to capture the rapidly changing hormone levels at this life stage and provides a strong foundation for studying the true impact of gonadal hormones on adolescent health, wellbeing, and behavior. It is important to quantify the health and behavioral implications of puberty hormones so as to avoid falsely over- or under-attributing characteristic adolescent mood and behavioral disturbances to “their hormones”; especially as these disturbances have potential lifelong consequences.

A similar study using serum samples would unlikely have been achievable, given the demonstrable challenges of recruiting a large community cohort of this age group [32] and considering the burden that repeated blood collections would place on young participants. Saliva as an alternate biological medium is prone to blood contamination; an issue that is not resolvable by mass spectrometry and is problematic especially in the early pubertal stage when detectable hormone levels are in the low ranges [50]. Our method for urinalysis has been developed and published [28, 29]. The urine values, higher than those in serum and thus easier to measure, correlate strongly with serum values [28]. Ultimately, the time-integrated nature of overnight urine collection is advantageous and more representative when compared with blood sampling, which captures hormone level at 1 instant in time. This is especially true of early puberty, during which gonadotropin activity is greatest at night [51]. The urine collection was also timed to the follicular phase in postmenarchal females.

A key and novel finding was the observation of 2 distinct urine hormone classes/trajectories in both sexes. These could be summarized in terms of stability of quarterly hormone change, together with a trend towards higher levels in those adolescents with the more unstable pattern. We have been unable to identify a study of similar sampling intensity in the literature. The majority of previous studies addressing this question have used less frequent and often randomly collected gonadal hormone samples in blood or saliva, or proxy markers such as anthropometry or Tanner stage. The latter physical measures are easy to perform but have recognized limitations [22].

Our analyses consistently identified 2-class GMMs as best fitting compared with 1- or 3-class models, regardless of centering value. Tanner stage 3 was chosen as a marker where rapid hormone upswings occur and when characteristic adolescent behaviors emerge. Substantial statistical power was lost with age at PHV centering as this was not a study of puberty onset. Centering on menarche is limited by its lateness in puberty and being a single-sex event. Thus, we believe that our data provide robust findings, given the analyses focused on relating hormone patterns to Tanner stage which reflects primarily gonadal hormone change, despite limitations around self-reporting. PHV is a mixed hormonal event occurring at clearly different Tanner stages and degrees of gonadal hormone rise in males and females [52]. Chronological age is also an inadequate indicator of pubertal progression given the wide range in onset [26, 27]. There were no easily identifiable anthropometric markers to delineate these 2 urinary hormone classes.

A second and intriguing finding was that peak urine estradiol levels in premenarcheal females may be as high as levels seen in menarchal females. Estradiol peaks prior to menarche indicate variably active follicular function in the ovaries from late childhood to early- to mid-puberty, supporting prior evidence from ultrasound and inhibin studies [53, 54]. Our data on menarchal onset are highly reliable, either provided by mothers in the small number of girls who had already reached menarche at recruitment, or recorded with the 3-monthly urine sample collections. One explanation for the premenarcheal estradiol peaks may be an intrinsic sex difference in the GnRH pulse regulator. These peaks may make hormone trajectories/patterns less clear in females. An example of this was our inability to replicate differences in TS progression observed between class 1 and 2 males.

In line with recent recommendations to expand scientific research on puberty [55], our findings afford the unique opportunity to test hypotheses concerning the relationship between gonadal hormone change and many commonly observed mood and behavioral changes in adolescence including mood lability, proneness to anxiety and depressive symptoms, disengagement with education, and increased health risk behaviors. It could be further hypothesized that the 2 identified trajectories of urine hormone change represent differences in gonadotropin-releasing hormone (GnRH) production patterns, although this cannot be confirmed on the available data.

The ARCHER study was never intended as a study of pubertal timing or onset, which has been well addressed by the impressive mixed cross-sectional/longitudinal Copenhagen Puberty Study [56], which used a lower biological sampling frequency. Rather, the intent of ARCHER was the detailed description of gonadal hormone change, over a sufficiently long period and in enough participants, to capture such change until the completion or near-completion of puberty. A limitation was the inability to conduct clinician staging of puberty as determined by our institutional Ethics Review Committee. In addition, urine collection over a longer duration would have enabled us to ascertain whether the 2 hormone trajectory classes remain with increasing gonadal maturity. We had neither resources nor adolescent consent or willingness to extend the intensive urine collection beyond 3 years.

In conclusion, a foundational study is presented which allows the exploration as to why some adolescents may be more affected by their puberty hormones than others. Other factors that need to be taken into account with such an exploration are the known neurocognitive development during adolescence [8, 57], when hyper-emotionality, risk-taking and reward-seeking are slowly modulated by ongoing maturation of the prefrontal cortex, as well as by exogenous factors such as family and peer influences. Our study has collected much data relevant to these considerations.

## Abbreviations

ARCHER: Adolescent Rural Cohort study of Hormones, Health, Education, Environments and Relationships
GMM: growth mixture model
GnRH: gonadotropin-releasing hormone
PHV: peak height velocity
SD: standard deviation
TS: Tanner stage

## Notes

***Financial support:*** The ARCHER study was funded by a grant from National Health and Medical Research Council of Australia (1003312). Additional support was received from the Thyne Reid Foundation, The Balnaves Foundation, Country Women’s Association of New South Wales and Sydney Medical School. Australian Rotary Health and the Sydney Medical School Foundation supported the initial feasibility studies.

***Clinical Trial Information:*** Australian New Zealand Clinical Trials Registry Number: ACTRN12617000964314

***Author contributions:*** KSS and DJH designed research; KSS, HLC, GML and DJH conducted research; DJH provided essential laboratory advice and materials; FLG, HLC, and KSS analyzed data; all authors interpreted data; KSS and FLG wrote paper; KSS had primary responsibility for final content; all authors read and approved the final manuscript.

## Additional Information

**Disclosure summary:** DJH has received institutional (but no personal) funding for investigator-initiated testosterone pharmacology studies and has provided expert testimony to anti-doping and professional standards tribunals as well as testosterone litigation. All other authors have nothing to disclose.
